# Oral Microbiota-Host Interaction Mediated by Taste Receptors

**DOI:** 10.3389/fcimb.2022.802504

**Published:** 2022-03-29

**Authors:** Hao Dong, Jiaxin Liu, Jianhui Zhu, Zhiyan Zhou, Marco Tizzano, Xian Peng, Xuedong Zhou, Xin Xu, Xin Zheng

**Affiliations:** ^1^State Key Laboratory of Oral Diseases and National Clinical Research Center for Oral Diseases, West China Hospital of Stomatology, Sichuan University, Chengdu, China; ^2^Department of Cariology and Endodontics, West China Hospital of Stomatology, Sichuan University, Chengdu, China; ^3^Stomatology Hospital, School of Stomatology, Zhejiang University School of Medicine, Hangzhou, China; ^4^Clinical Research Center for Oral Diseases of Zhejiang Province, Key Laboratory of Oral Biomedical Research of Zhejiang Province, Cancer Center of Zhejiang University, Hangzhou, China; ^5^Basic and Translation Sciences, Penn Dental Medicine, University of Pennsylvania, Philadelphia, PA, United States

**Keywords:** taste receptor, oral microbiota, innate immunity, periodontitis, dental caries, diet

## Abstract

Taste receptors, originally identified in taste buds, function as the periphery receptors for taste stimuli and play an important role in food choice. Cohort studies have revealed that single nucleotide polymorphisms of taste receptors such as T1R1, T1R2, T2R38 are associated with susceptibility to oral diseases like dental caries. Recent studies have demonstrated the wide expression of taste receptors in various tissues, including intestinal epithelia, respiratory tract, and gingiva, with an emerging role of participating in the interaction between mucosa surface and microorganisms *via* monitoring a wide range of metabolites. On the one hand, individuals with different oral microbiomes exhibited varied taste sensitivity, suggesting a potential impact of the oral microbiota composition on taste receptor function. On the other hand, animal studies and *in vitro* studies have uncovered that a variety of oral cells expressing taste receptors such as gingival solitary chemosensory cells, gingival epithelial cells (GECs), and gingival fibroblasts can detect bacterial signals through bitter taste receptors to trigger host innate immune responses, thus regulating oral microbial homeostasis. This review focuses on how taste receptors, particularly bitter and sweet taste receptors, mediate the oral microbiota-host interaction as well as impact the occurrence and development of oral diseases. Further studies delineating the role of taste receptors in mediating oral microbiota-host interaction will advance our knowledge in oral ecological homeostasis establishment, providing a novel paradigm and treatment target for the better management of dental infectious diseases.

## Introduction

Taste is triggered by signals from the oral sensory structures known as taste buds, which are stimulated by tastants and then conveyed to the central gustatory nervous system (Small, 2012; von Molitor et al., 2021). Various tastes including sweet, bitter, umami, sour, and salt are mainly perceived by specific taste receptors located at taste buds, which help the host to discriminate between nutrients and poisonous and harmful substances (Margolskee, 1993; Roper, 2013). General health may be impaired by dysfunctional or diseased states of taste receptors. Past studies have well illustrated that gustatory sensitivity is influenced by single nucleotide polymorphisms (SNPs) of taste receptors and leads to different food preferences, which contribute to varied oral microbiota and disease susceptibility in the host (Chamoun et al., 2018b).

Recent studies have found that taste receptors are not only distributed in cells within taste buds, but are also expressed on a variety of cell types both orally and extra-orally, such as tuft cells, airway smooth muscle cells, macrophages, and so on (Deshpande et al., 2010; Sbarbati et al., 2010; Grassin-Delyle et al., 2019). Meanwhile, studies on taste receptors both on and off the taste buds support the view that taste receptors play an essential role as chemoreceptors in diverse non-gustatory physiological and pathological processes (Lee et al., 2014; Schneider et al., 2019; Gopallawa et al., 2021). Taking the oral taste receptors as an example, on the one hand, taste receptors detect various metabolites and other toxins derived from the oral microbiota, contributing to the establishment of the host immune response and the maintenance of homeostasis (Gil et al., 2015; Zheng et al., 2019; Medapati et al., 2021b). On the other hand, the perceptive capacity of taste receptors is in turn shaped by the oral microbiota (Solemdal et al., 2012; Zhu et al., 2021). Studies in other tissues have yielded similar findings (Tizzano et al., 2010; Deckmann and Kummer, 2016). The investigation of how taste receptors are involved in the interaction between host and oral microbiota will facilitate a thorough understanding of the chemosensory function of taste receptors, which is of great significance for the elucidation of the association between taste receptors and oral diseases. Accordingly, the role of taste receptors in regulating oral health and disease states will be discussed in this review, with a focus on the impact of genotypic and phenotypic changes in receptors on the composition of the oral microbiota, as well as the key role of taste receptors mediating innate immune responses in oral microbiota-host interaction.

## Molecular Mechanisms of Taste Signal Transduction

Taste receptors are chemosensory receptors that exist in both taste buds and extra-gustatory tissues (Chandrashekar et al., 2006; Carey and Lee, 2019). Five widely accepted and fundamental tastes (sweet, umami, salt, sour, bitter) are initiated from these taste receptors (Lindemann, 2001). In recent studies, kokumi and fat are likely to be potential candidates for new basic tastes (Khan et al., 2019; Rhyu et al., 2020; Hichami et al., 2021). The receptors, including multiple members of the G protein-coupled receptor (GPCR) superfamily and some ionic channels, put different specificity to stimuli (Nuemket et al., 2017; Teng et al., 2019; Ahmad and Dalziel, 2020). In practical terms, sweet means consuming carbohydrates as vital energy source, salt indicates ingestion of sodium, and umami promotes detecting amino acids, which are respectively indispensable for energy metabolism, ionic homeostasis and building proteins (Yamaguchi and Ninomiya, 2000; Dias et al., 2012; Laffitte et al., 2014). Sour, which perceives rotten food, and bitter, which implicate a variety of toxic substances such as alkaloids and cyanogenic glycosides, are revolting tastes (Glendinning, 1994; Huang et al., 2008). Therefore, sour and bitter tastes promote the establishment of early warning against the intake of underlying toxins.

**Figure 1:**
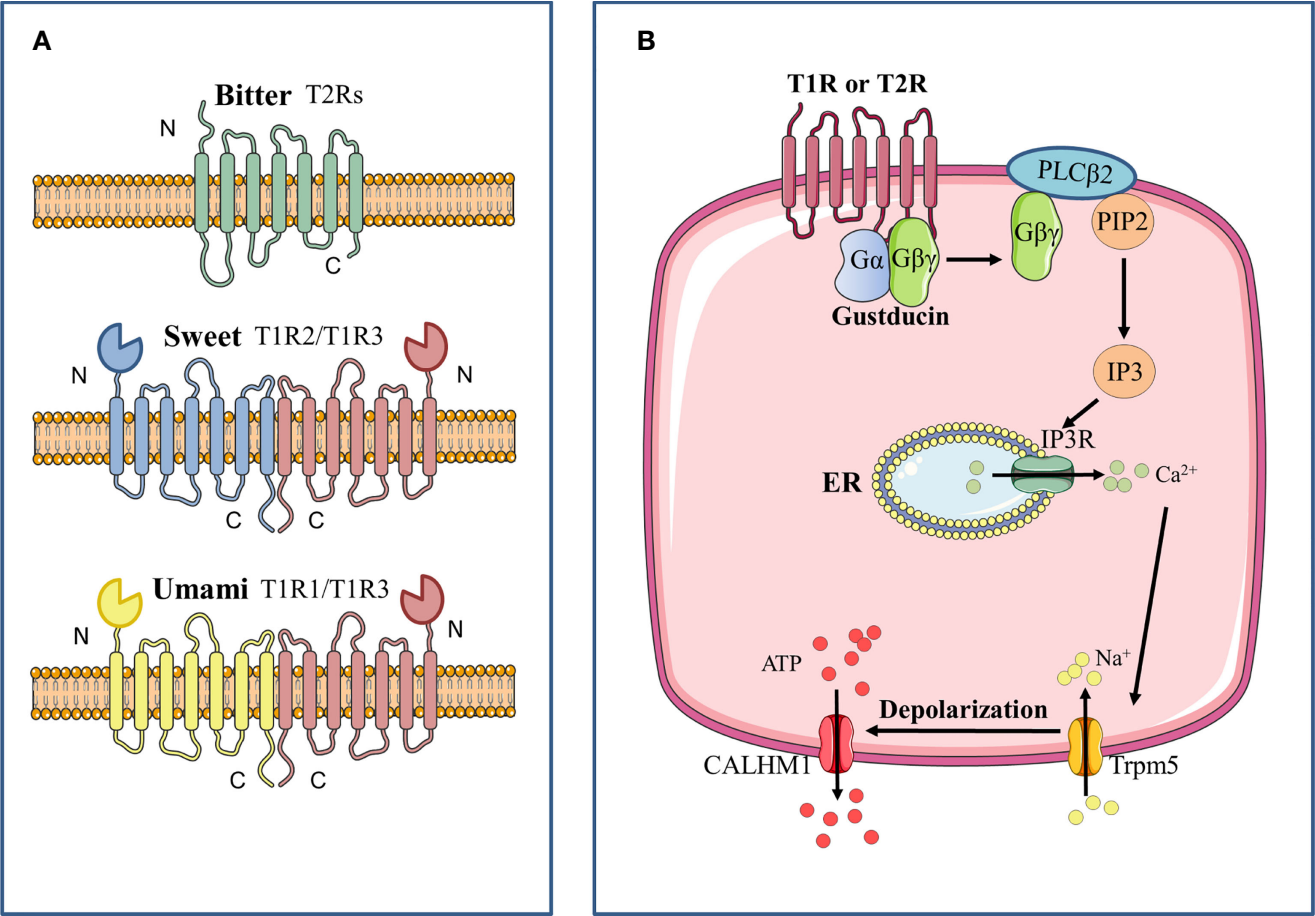
Signal transduction pathway of bitter, sweet, and umami GPCRs. **(A)** Bitter, sweet and umami receptors are all G-protein coupled-receptors. Bitter receptors are composed of T2Rs, while sweet (T1R2/T1R3) and umami (T1R2/T1R3) receptors are composed of T1Rs, which are characterized by a large N terminal domain that forms a Venus flytrap structure. **(B)** After stimulation of the taste receptor, the downstream Gβγ complex is mobilized, which then activates phospholipase C isoform β2 (PLCβ2) to induce the production of inositol 1,4,5-trisphosphate (IP3). IP3 then activates the IP3 receptor (IP3R), an intracellular ion channel that allows the release of Ca^2+^ from the endoplasmic reticulum (ER), resulting in an increase in intracellular Ca^2+^. The complex of transient receptor potential cation channel subfamily M member 5 (TRPM5) is then activated and triggers the inward Na^+^ diffusion. The depolarization causes activation of the complex of calcium homeostasis modulator 1 (CALHM1) channels, thus resulting in the release of ATP as the neurotransmitter.

Bitter, sweet and umami taste signals are supposed to converge on a common intracellular signaling transduction in the Type II cells. Bitter, sweet, umami tastes are activated by GPCRs, which are seven-transmembrane proteins of two main classes. GPCRs that detect sweet and umami stimuli are named as taste receptor family 1 member (T1R) (Zhao et al., 2003), and those that transduce bitter compounds are named as taste receptor family 2 member (T2R) (Chandrashekar et al., 2000). When activated by corresponding stimuli, G protein coupled to these taste receptors is resolved into G_βγ_ subunit and G_α_ subunit including G_α_-gustducin, G_α14_ and G_αi_ (McLaughlin et al., 1992; Huang et al., 1999; Tizzano et al., 2008). With further hydrolysis of G_βγ_, G_β3_ and G_γ13_ can be formed, which stimulates phospholipase Cβ2 (PLCB2) to increase intracellular Ca^2+^ levels (Zhang et al., 2007). G_α_ subunit is considered to influence cyclic adenosine monophosphate (cAMP) signaling (Clapp et al., 2008). When bitter and umami receptors are activated by tastants, G_α_ subunit diminishes intracellular cAMP levels, which inhibits activity of protein kinase A. As a result, it weakens the inhibition of protein kinase A on the PLCB2-IP_3_ pathway, and further promotes the release of Ca^2+^ in the endoplasmic reticulum. When sweet receptors are activated by tastants, G_α_ subunit increases intracellular cAMP levels, which enhances the activity of protein kinase A and inhibits K^+^ channels, thereby promoting extracellular Ca^2+^ influx. At last, elevated intracellular Ca^2+^ levels promote the opening of transient receptor potential cation channel subfamily M member 5 (TRPM5), which is an ion channel that results in membrane depolarization and causes action potential followed by the release of ATP (**Figure 1**) (Iwata et al., 2014).

Sour taste receptors are located in a subset of taste receptor cells, the Type III cells, on the tongue and palate epithelium (Chandrashekar et al., 2006). Recent studies have demonstrated that sour taste is initiated by a proton-selective channel Otopetrin-1(OTOP1), which is a 24 transmembrane protein (Tu et al., 2018; Zhang et al., 2019). Up to date, OTOP1 and other otopetrin family members are supposed to be the only voltage-insensitive and proton-selective ionic channels (Teng et al., 2019), which may explain how Type III cells are activated by small changes in proton levels and tolerate much more massive changes in other ion levels when in food intake. The increasing intracellular proton levels mediated by OTOP1, on one hand, proximately change the membrane potential (Zhang et al., 2019), on the other hand, subsequently block K_IR_2.1, a member of inward-rectifier K^+^ channels, to generate membrane depolarization (Ye et al., 2016). However, how OTOP1 gates and passes through protons and whether there are other elements in sour taste transduction remain to be solved in the future.

Salty taste in most mammals is divided into two pathways: appetitive salt taste and high-salt taste. Animals and humans adapt easily to appetitive salty taste in a low concentration (<100 mM) (Heck et al., 1984). This appetite is likely to meet the host requirement of Na+ to maintain an ionic homeostasis. By comparison, high-salt taste (>300 mM) is significantly aversive (Oka et al., 2013), which indicates a reflex that protects individuals against hypernatremia and dehydration. Appetitive salt taste is activated by epithelial sodium channels (ENaC), a heterotrimer consisted of α, β and γ subunits (Canessa et al., 1994), in some taste cells within fungiform papillae (Nomura et al., 2020). With the access of Na^+^ through ENaC, it causes a membrane depolarization driving action potentials. Notably, there is no change in intracellular Ca^2+^ levels with the generation of action potentials in the ${\text{ENaC}}_{\alpha }^{+}$ taste cells. ATP is released by calcium homeostasis modulator 1 (CALHM1) and CALHM3 subunits which are switched on under depolarization. Some studies have indicated that activation of s high-salt taste is associated with some types of bitter and sour taste cells in the foliate papillae and circumvallate papillae (Kretz et al., 1999; Lewandowski et al., 2016). However, there are no specific receptors and well-defined salty transduction signaling that have been demonstrated to function with high-salt taste.

## Genetic Variation of Taste Receptors Correlated With Oral Diseases

Varied dietary patterns among individuals are influenced by differences in taste perception abilities, thus shaping different oral health status. Genetic variations in certain genes associated with sweet, bitter, umami, salt, and sour tastes have been shown to correlate with taste function in varying degrees, and are thought to be potentially relevant affectors of oral disease susceptibility (**Table 1**) (Chamoun et al., 2018b).

**Table 1:** SNPs of taste receptors correlated with oral diseases.

	Gene	SNP ID	Outcome	Reference
Sweet	TAS1R2: 18854899T > C	rs35874116	Individuals with Ile191Val consumed fewer sugars as well as faced a lower risk of developing dental caries compared with Ile homozygotes.	(Eny et al., 2010; Kulkarni et al., 2013; Haznedaroğlu et al., 2015)
			The Ile/Val and Ile/Ile genotypes appeared with a lower carbohydrate intake compared with the Val/Val genotype among the population of West Mexico.	(Ramos-Lopez et al., 2016)
			Children with Ile191Val were more frequently affected by caries than the common Ile allele.	(Izakovicova Holla et al., 2015)
	TAS1R3: -1572C > T	rs307355	TAS1R3 gene rs307355 polymorphism has been found to be an independent risk factor for dental caries experience and to have increased the risk of caries.	(Haznedaroğlu et al., 2015)
Bitter	TAS2R38: 145G > C (A49P) 785T > C (V262A) 886T > C (I296V)	rs713598 rs1726866 rs10246939	The PAV (taster) haplotype was protective against dental caries.	(Wendell et al., 2010)
			PROP non-tasters presented with significantly increased caries risk than PROP tasters.	(Öter et al., 2011)
			PAV/PAV homozygosity had the strongest ability to induce T2R38 expression when stimulated by *S. mutans* whereas AVI/AVI changed little.	(Gil et al., 2015)
Umami	TAS1R1: 6576401C > T	rs17492553	“Super-tasters” CC homozygotes tend to have a lower risk of dental caries prevalence.	(Rawal et al., 2013)

### Sweet Taste Receptors

The perception of sweet taste is mediated by a heterodimer receptor composed of T1R2 and T1R3 together (Li et al., 2002). Various kinds of sweet taste compounds are detected by the T1R2/T1R3 sweet taste receptor, such as natural sugars, nonnutritive sweeteners, sweet-tasting proteins, and so on (Chandrashekar et al., 2006). The activation of the sweet taste receptor initiates intracellular signal transduction, mediating the regulation of the production and secretion of physiologically significant hormones and proteins like insulin and glucagon-like peptide-1 (GLP-1) (Kojima and Nakagawa, 2011; Kyriazis et al., 2012). Moreover, the sweet taste is a source of hedonic liking and greatly influences people’s dietary choices (Jayasinghe et al., 2017). Disruption or loss of sweet taste receptor function would cause a wide range of health problems including metabolic disorders and oral diseases (Murovets et al., 2015; Smith et al., 2016).

Specifically, differences in the capacity of sweet taste receptors are associated with their genetic variation (Garcia-Bailo et al., 2009). SNPs of T1R2 and T1R3 determine sweet taste perception thresholds and the degree of sweet food preference and consumption (Jayasinghe et al., 2017). Eny et al. pointed out Ile191Val (rs35874116) variations in T1R2 were associated with different sugar intake, as Val allele carriers consumed fewer sugars compared with the Ile homozygotes (Eny et al., 2010). A recent study conducted in a Mexican population reported that a higher carbohydrate intake, as well as the risk of hypertriglyceridemia (HTG), was found in the Val/Val genotype individuals versus the Ile/Val and Ile/Ile genotypes (Ramos-Lopez et al., 2016). In addition, the studies of T1R3 revealed the association between SNPs and sweet taste sensitivity as well (Fushan et al., 2009; Murovets et al., 2020).

As aforementioned, SNPs of sweet taste receptors lead to differences in dietary sugar intake (Garcia-Bailo et al., 2009). The ingested carbohydrates are metabolized to produce acid in the oral biofilm made up of microorganisms that adhere to the tooth surface (Bradshaw and Lynch, 2013). When the pH of the tooth surface falls below 5.5, minerals are lost from the tooth surface faster than remineralization, which ultimately leads to dental caries. Put simply, sweet taste receptor gene polymorphisms shape the risk of caries by altering sweet food preferences. Studies conducted by Kulkarni et al. and Haznedaroglu et al. concluded that individuals who hold an Ile191Val polymorphism consumed fewer sugars with a lower risk of developing dental caries, while Ile homozygotes whose carbohydrate intake are higher showed more frequent high-risk caries experience (>8 caries) (Kulkarni et al., 2013; Haznedaroğlu et al., 2015). These conclusions are consistent with most of the other studies (Eny et al., 2010; Ramos-Lopez et al., 2016). However, one study reported an opposite finding that children who carried the Val allele were more frequently affected by caries than the common Ile allele (Izakovicova Holla et al., 2015). One possible explanation is that differences in race, environment, and food culture among populations are also involved in the effect of sweet taste receptor SNPs on dietary choices as well as the risk of caries. Another possibility is that the different genotypes of taste receptors not merely determine the sensitivity of taste perception, but are also involved in the oral microbiota-host interaction through other mechanisms.

Additionally, the increased intake of sugar caused by T1R2/T1R3 SNPs also modifies the oral microecological environment, leading to the alteration of the composition of oral microflora. A recent study revealed that children with low sensitivity to sweet taste presented with a higher incidence of dental caries mainly due to their more frequent consumption of sweet food, who also appeared to contain an increased presence of cariogenic *Streptococcus mutans* (*S. mutans*) (Jurczak et al., 2020). Altered microbial diversity occurs after a sugar-rich diet, which greatly increases the presence of cariogenic species, mediating the further development of caries (Tanner et al., 2018).

### Bitter Taste Receptors

Human detects numerous different bitter tastes through 25 types of T2Rs, while T2R38 plays the most vital role that genetically controls the perception capacity of bitter taste (Kim et al., 2003; Dong et al., 2009). Bitter taste receptors often work in conjunction with sweet taste receptors to influence our diet. Simply as people with high sweetness sensitivity reduce their intake of sweet food, people with high bitter sensitivity often tend to avoid bitter food. However, reduced intake of bitter foods such as antioxidant-rich vegetables and fruits may lead to a higher risk of cardiovascular disease or cancer (Basson et al., 2005; Roura et al., 2016).

The bitter taste sensation of agonists PTC and PROP is mediated by their combination with T2R38 (Kim et al., 2003; Bufe et al., 2005). Three SNPs of T2R38 caused by different combinations of amino acid substitutions respectively at positions 49 (Pro49Ala, rs713598), 262 (Ala262Val, rs1726866), and 296 (Val296Ile, rs10246939) mainly regulated the diverse senses of bitter taste (Kim et al., 2003). Homozygous for the PAV haplotype are characterized by a stronger perception of bitterness than the average person and are called “super-tasters”, while those homozygous for the AVI haplotype manifest as “non-tasters” whose sense of bitter taste are less sensitive. The performance of heterozygotes is intermediate (Bartoshuk et al., 1994).

Various studies have confirmed that some undesirable diet may be attributed to the “supertaster” phenotype and have further linked the effects of bitter taste receptor SNPs leading to food preferences to health-disease transformation. Wendell et al. reported that the PAV (taster) haplotype is proven to be protective against dental caries (Wendell et al., 2010). The result of a subsequent study by Öter et al. confirmed this surprising view, as PROP non-tasters presented with significantly increased caries risk than PROP tasters (Öter et al., 2011). Interestingly, the capacity of T2R38 expression stimulated by *S. mutans* exhibits a polarity. PAV/PAV homozygosity had the strongest ability to induce T2R38 expression whereas AVI/AVI changed little (Gil et al., 2015). However, the theory that taste receptor mediates food selection fails to explain this phenomenon, as “super-tasters” have a stronger perception of bitterness and tend to consume less healthful bitter foods, yet they have a lower risk of caries and a lower abundance of pathogenic bacteria. This suggests the presence of pathogenic factors independent of taste receptor-mediated food selection. Further studies are needed to elucidate the pathogenesis involved.

In addition, several bitter taste receptors other than T2R38 have recently been shown to be as well involved in interactions with the oral flora and induction of disease. A recent study uncovered the novel role of bitter taste receptor T2R14 in detecting *S. mutans* and mediating innate immune defense in GECs (Medapati et al., 2021b). Moreover, CA6 gene polymorphisms were associated with *S. mutans* colonization, oral microbiota composition and the risk of dental caries, which were linked to bitter taste and smell perception (Esberg et al., 2019).

### Other Taste Receptors

Umami taste is initiated and enhanced through the heterodimer T1R1/T1R3 (Zhao et al., 2003). The primary umami taste substance is free l-glutamate, a compound that we are familiar with its another form called monosodium glutamate (MSG) (Ikeda, 2002). Two SNPs caused the substitutions of amino acid in the T1R1 respectively at positions 110 (Ala110Val, rs41278020) and 372 (Ala372Thr, rs34160967) are associated with sensitivity in umami taste (Raliou et al., 2009). An intronic SNP (rs17492553) in T1R1 is related to taste intensity in sweet, bitter, salty and sour (Rawal et al., 2013). CC homozygotes appeared to possess stronger taste intensity as “super-tasters” than that in TT homozygotes from rs17492553 groups. Similar to the trend in rs17492553 that in rs34160967, GG groups are stronger in overall taste intensity than that in AA/AG groups (Rawal et al., 2013). A recent study conducted in a Brazilian pre-adolescent population has shown that rs17492553 in T1R1 was relevant to dental caries prevalence. “Super-tasters” CC homozygotes tend to have a lower risk of dental caries prevalence. The impact of SNPs in other taste receptors such as ENaC and OTOP1 on taste perception and oral disease susceptibility has remained obscure.

## Regulation of Taste Receptors on Oral Microbiota-Host Interaction

Since its discovery, the associations between taste receptors and oral diseases have been extensively studied. The prevailing notion is that the SNPs of taste receptors enable the host to perceive various tastes differently, which influenced dietary choices and host oral microbiome, regulating the host’s metabolism along with internal environmental homeostasis and ultimately mediating the reciprocal transition between health and disease states. Meanwhile, as we noted previously, food selection conducted by taste receptors SNPs fails to explain the full range of disease events. Several recent studies have focused on revealing how the taste receptor acts as a chemoreceptor to engage in signal transduction and immune responses in the oral cavity, which provides fresh insights into the role that taste receptors play in oral diseases. The latest relevant advancements are presented and summarized below, providing fresh perspectives on how taste receptors interact with oral microbiota to manage oral health status.

### Taste-Shaped Diet Impacts Host Metabolism and Microbial Homeostasis

As previously mentioned, the differential perceptive capacity of taste receptors due to SNPs influences individual food preferences. Recent studies have confirmed that taste preferences for sweet, bitter, sour and salt are affected genetically in part and have an impact on food and beverage choices (Chamoun et al., 2018a; Eriksson et al., 2019; Chamoun et al., 2021). Ample research evidence suggests that diet is one of the most important factors influencing human health and that different dietary patterns greatly regulate host metabolism and microbial homeostasis. For example, a high-fat diet in healthy adults leads to altered gut microbiota, including higher levels of *Alistipes* and *Bacteroides* species and a decrease in *Faecalibacterium* species, as well as increased faecal metabolites p-resol and indole, which are implicated in a higher risk of cardiovascular and metabolic disturbances (Wan et al., 2019). Some researchers have also suggested that microbiota can in turn alter dietary patterns by manipulating host taste perception (Cattaneo et al., 2019b). Overall, taste perception could be involved in host-microbiota interaction by influencing dietary patterns.

Further, impairment or loss of taste function poses a systemic and multilayered risk to people, rather than solely oral diseases. As a consequence of altered taste perception, many foods may be averted owing to the difficulty in appreciating the diet, thus shifting people towards unhealthy eating habits and potentially leading to serious consequences (Chamoun et al., 2018b). For instance, an increase in salt taste recognition threshold may lead people to consume more salt to improve food palatability, thereby elevating their risk of cardiovascular diseases (Pilic and Mavrommatis, 2018; Tapanee et al., 2021). Notably, a national health survey in the US reported that participants who met the recommendations of the Healthy Eating Index (HEI-2015) scores were at a lower risk of developing untreated coronal caries than those who did not conform to the recommendations (Kaye et al., 2020). In addition, shifted taste perception has also been proven to be correlated with depression and anxiety (Esposito et al., 2021).

In addition, taste receptors are associated with the regulation of body metabolism and internal environmental homeostasis. Several studies have revealed the secretory function of taste receptors in the gastrointestinal tract. For example, TAS2Rs regulate intestinal anion secretion to protect the host from ingesting dietary toxins (Glendinning, 1994). Smith et al. proved that intestinal sweet taste receptors modulate gut hormone responses and glucose absorption, as glucose-dependent stimulation of intestinal T1R2/T1R3 chemo-sensors on L-cells enhances glucose absorption through GLP-2-mediated activation of GLUT2 transporter in enterocytes (Smith et al., 2018). Serrano et al. subsequently found that T1R2 gene variant Ile191Val causes a partial loss of function and results in reduced glucose excursions during an OGTT, which is independent of the variations in beta-cell function or insulin sensitivity (Serrano et al., 2021). Additionally, bitter taste receptors have also been revealed of the function to evoke airway smooth muscle relaxation *via* localized calcium flux, predicting the potential role in counteracting asthmatic bronchoconstriction (Deshpande et al., 2010). Recent studies have also found that older carriers of variant rs236514 (A) of the *KCNJ2* gene, which is located in Type III sour-sensing taste cells and affects sour perception thresholds, have a higher preference for acid and lower energy intake, along with an increased risk of mild-to-severe cognitive impairment (Chamoun et al., 2018b; Ferraris et al., 2021a; Ferraris et al., 2021b).

### Microbiota Modulate the Perceptive Capacity of Taste Receptors

Significant relationships between oral microbiota composition and taste sensitivity were found recently. A study conducted by Solemdal et al. in acutely hospitalized elderly showed reduced taste ability in patients with poor oral hygiene such as caries activity, among whom patients with high *Lactobacillus* growth had the most significant impairment in sour taste (Solemdal et al., 2012). This result may be in relation to the adaptation of sour taste perception due to acid produced by bacteria and the increased sour taste threshold. Cattaneo et al. revealed a significant difference among subjects with varied responsiveness to PROP in terms of the relative abundance of some taxa (Cattaneo et al., 2019a). Subjects characterized by greater PROP responsiveness showed overrepresentation mainly in five bacterial genera, including *Actinomyces*, *Oribacterium*, *Solobacterium*, *Catonella* and *Campylobacter*. Their further study also found that specific bacterial taxa mainly composed of *Clostridiales* and *Bacteroidales* may influence host sensitivity to salt and acid, as these taxa were negatively correlated with perceived taste thresholds for salt and acid (Cattaneo et al., 2019b). Similarly, Feng et al. reported that elevated proportions of *Actinomyces* and *Firmicutes* in the saliva were associated with reduced taste sensitivity, whereas increased taste sensitivity resulted from higher proportions of *Bacteroides* in the tongue film (Feng et al., 2018). Besides, a study by Besnard et al. demonstrated that the low-fat tasters showed greater oral bacterial diversity and a high *Bacteroides/Lactobacillus* ratio which significantly promotes host inflammation compared to the high-fatty tasters (Besnard et al., 2020). Impairment of oral fat perception may form a feedback loop with such a pro-inflammatory bacterial community composition, as obesity-induced inflammation has been shown to damage taste buds in mice and reduce fatty taste sensitivity in human (Jilani et al., 2017; Kaufman et al., 2018). Our latest study as well illustrated that bacterial lipopolysaccharide-induced alternative splicing of the mouse sweet taste receptor T1R2, thus contributing to a significant upregulation of its non-functional isoform expression and inhibition of sweet taste perception (Zhu et al., 2021). It can be hypothesized that these impaired or non-functional isoforms of taste receptors would similarly lack the ability to detect and mediate the elimination of pathogens.

There are several possible interpretations of the perceptive changes in taste receptors owing to the growth of specific oral bacteria. One possibility lies in the ability of oral bacteria to modulate the food preferences of the host. Microbes in the gastrointestinal tract have shown a potential role in manipulating host diet by regulating chemoreceptor expression (van de Wouw et al., 2017). This is in line with the recent study which indicated that higher relative abundance of oral *Clostridia* is associated with increased total energy, fat and protein intake as well as reduced fiber consumption, while *Proteobacteria phylum* and *Prevotella genus* exhibited the reverse correlation (Cattaneo et al., 2019b). Another plausible explanation rests on the fact that bacterial metabolites of the compounds can activate or modulate host taste perception. For instance, host sensitivity to sucrose is correlated with the sucrose catabolism of different oral bacteria *in vivo* (Gardner et al., 2020; Sedghi et al., 2021). Some specific intraoral bacteria like *Porphyromonas gingivalis* (*P. gingivalis*) are capable of utilizing salivary glutamates so as to modulate their concentration, ultimately affecting the taste perception (Takahashi, 2015). Consistent findings were observed in investigations that examined the impacts of different oral microbiota on the metabolism and aroma perception of cysteine conjugates as well as glycosides (Starkenmann et al., 2008; Parker et al., 2020). Furthermore, oral microbiota also modulates host chemosensation by altering taste receptor density, which is attributed to the ability of certain microorganisms to mediate the secretion of inflammatory factors by host cells and trigger inflammation. On the one hand, the immune response can diminish taste perception through damaging taste buds (Wang et al., 2009; Cohn et al., 2010). On the other hand, several studies have also demonstrated that taste receptors themselves have varying degrees of responsiveness to microorganisms and are directly engaged in the host immune regulation (Gil et al., 2015; Zheng et al., 2019). We elaborate below on the latest progress in studying the participation of taste receptors in the host immune response.

### Taste Receptors Implicated in the Host Immune Response to Microorganisms

**Figure 2:**
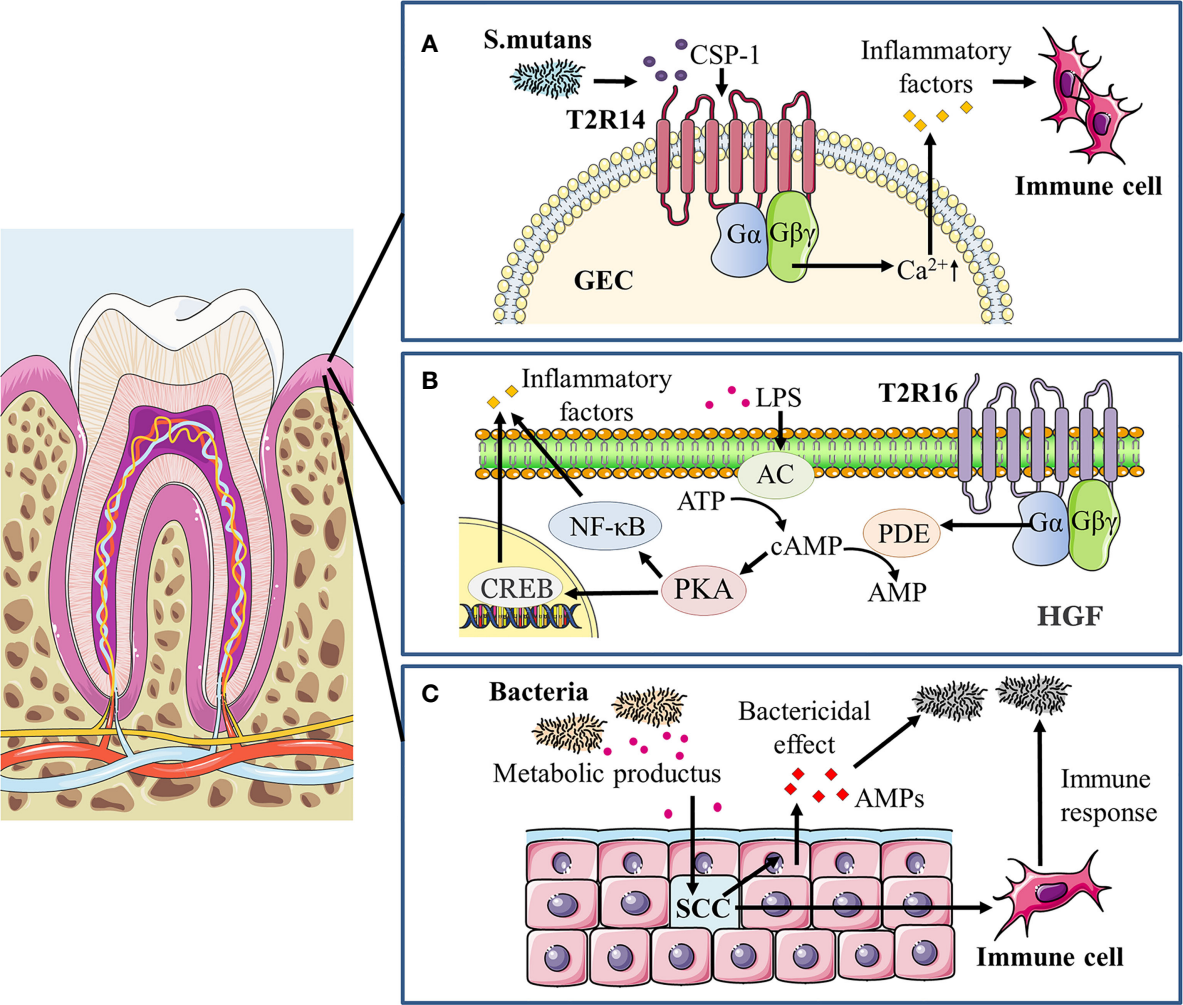
Taste receptors modulate oral immune response to microorganisms. **(A)** In gingiva epithelial cells (GECs) treated with *S. mutans* competence stimulating peptide-1 (CSP-1), a vigorous increase in intracellular calcium mobilization and secretion of cytokines/chemokines including IL-6, IL-8 and TNF-α occurred primarily through the T2R14-Gβγ-PLCβ pathway, recruiting immune cells and mediating the immune response to pathogens. **(B)** LPS can induce a significant dose- and time-dependent increase in adenylate cyclase (AC) activity and thereafter elevates intracellular cAMP levels, positively affecting NF-κB activity through its major effector protein kinase A (PKA) and stimulating inflammatory responses. In addition, the cAMP/PKA/cAMP response element binding protein (CREB) signaling pathway may promote the LPS-induced release of pro-inflammatory cytokines, including IL-6, IL-33, and TNF-α. In human gingival fibroblasts (HGFs), agonist-stimulated T2R16 mobilizes downstream α-gustducin to activate the phosphodiesterase (PDE) that hydrolyzes cAMP, thereby reducing intracellular cAMP levels and alleviating the LPS-induced inflammation as well as tissue injury. **(C)** Taste receptors of gingival solitary chemosensory cells (gSCCs) activated by bacterial metabolites would stimulate epithelial cells to release antimicrobial peptides (AMPs) as a direct bactericidal effect and also recruit immune cells to modulate the oral immune response.

As previously stated, taste receptors act as chemoreceptors that not only sense chemicals known as tastants to initiate taste signals, but also recognize microorganisms such as bacteria and activate downstream signaling cascades, thereby contributing to innate host defense and the maintenance of microbial homeostasis (Lee et al., 2014; Gil et al., 2015; Jaggupilli et al., 2018). In the past, great progress has been made on the involvement of taste receptors in extraoral organs such as airways and gastrointestinal tract in host innate immunity and microbial regulation, while the role of taste receptors in the oral cavity in mediating microbiota-host interaction is minimally known. A number of recent studies have yielded new light on oral taste receptors that detect bacterial signals and subsequently coordinate immune responses (**Figure 2**).

Taste receptors have been detected to be expressed on a variety of tissues and cells, and are known to be engaged in host immunity (Workman et al., 2015; Bloxham et al., 2020; Welcome, 2020). Expression of bitter receptors is observed in immune cells such as monocytes and neutrophils, which aids in the recognition of bacterial quorum sensing molecules (QSMs) and facilitates the innate immune response to disease (Maurer et al., 2015). T2Rs (mainly T2R38 and T2R14) in airway epithelial cells specifically bind to the *Pseudomonas aeruginosa* derived QSMs acyl homoserine lactones (AHLs) and initiates the host immune response, activating chemotaxis of immune cells and promoting the secretion of antimicrobial peptides (AMPs) as well as inflammatory factors (Hariri et al., 2017; Freund et al., 2018; Jaggupilli et al., 2018). In addition, the mechanism of T2R participation in the immune response has been further revealed by the work of Gopallawa et al. in macrophages (Gopallawa et al., 2021). Bitter taste metabolite from bacteria stimulates bitter taste receptors and activates calcium signaling, leading to activation of nitric oxide synthase (NOS) isoforms with nitric oxide (NO) production. NO may upregulate phagocytosis of macrophages *via* several mechanisms (Jun et al., 1996). Simultaneously, bacterial co-stimulation of macrophages and T2Rs also enhanced phagocytosis by reducing cAMP when cAMP levels were elevated from baseline. Interestingly, sweet taste may regulate innate immunity in reverse to bitter taste signals. Lee et al. demonstrated that T1R2/3 receptors activated by sugars or bacterial d-amino acids could inhibit T2R-dependent calcium signaling and downstream AMP secretion of adjoining epithelial cells, diminishing the immune response to microbial bitter products (Lee et al., 2014; Lee et al., 2017). Furthermore, T2Rs activation can also induce anti-inflammatory effects as antagonizing lipopolysaccharide (LPS)-provoked production of inflammatory mediators in human peripheral mononuclear blood cells and lung macrophages (Tran et al., 2018; Grassin-Delyle et al., 2019). The anti-inflammatory activity of T2Rs was likewise associated with SNPs, as the functional PAV haplotype T2R38 receptor exhibited significant inhibition of TNF-α release after agonist stimulation compared to the non-functional AVI/AVI diplotype (Tran et al., 2018).

Studies in the oral cavity have expanded the role of taste receptors, especially bitter taste receptors, in the recognition of oral pathogenic microorganisms and modulation of oral microbial homeostasis. Gil et al. showed that the T2R38 gene polymorphism in the oral cavity determined the degree of innate immune response induced by oral bacteria (Gil et al., 2015). T2R38 mRNA expression in GECs carrying PAV/PAV genotype increased significantly when stimulated with *S. mutans*, while AVI/AVI genotype showed little change. Human β-defensin-2 (hBD-2) and IL-1α secretion after the stimulation of *S. mutans* was decreased in PAV/PAV cell line after the T2R38 knockdown. The result agrees with Wendell et al. who reported that the PAV haplotype of T2R38 exhibited a protective effect against caries in primary dentition, whereas the AVI haplotype posed a higher risk of suffering from caries (Wendell et al., 2010). In the same study by Gil et al., an opposite result was found for stimulation from *P. gingivalis* or *Fusobacterium nucleatum*, as the expression of T2R38 was significantly upregulated in the GECs of AVI/AVI genotype, while no significant changes were observed in PAV/PAV or PAV/AVI genotype (Gil et al., 2015). This suggests that different pathogenic bacteria may act through different signaling pathways to stimulate taste receptors and elicit subsequent immune responses in the organism. The study by Medapati and his colleagues then uncovered an emerging role of T2R14 in recognizing oral *S. mutans* with activation of innate immune response in GECs (Medapati et al., 2021b). T2R14 in GECs mediates calcium signaling and secretion of pro-inflammatory cytokines upon recognition of competence stimulating peptide-1 (CSP-1) secreted by *S. mutans*, as well as attracting differentiated HL-60 immune cells (dHL-60). A subsequent study by Medapati et al. further pointed out that T2R14 may mediate the internalization of *Staphylococcus aureus* (*S. aureus*) in GECs through activating p21-activated kinase 1 associated actin and F-actin, and enhanced hBD-2 secretion that inhibits *S. aureus* (Medapati et al., 2021a). However, the knockdown of T2R14 alone does not affect the level of the internalization of *S. mutans* and the secretion of hBD-2 in GECs. It is interesting to observe that T2R14-dependent inhibition of *S. aureus* occurred when GECs were treated with CSP-1 from *S. mutans*. This hints that bitter receptor-mediated bactericidal effects may be one of the important mechanisms by which *S. mutans* overcome competition with commensal bacteria in the oral cavity. Of note, study evidence also suggests that bitter taste receptors may similarly assume a repressive role against inflammation development in the oral cavity. Our recent work unveiled that the bitter agonist salicin decreased LPS-induced cAMP accumulation in human gingival fibroblasts in a T2R16-dependent manner, and subsequently suppressed the expression of inflammatory cytokines along with NF-κB activation (Zhou et al., 2021). The simultaneous function of T2Rs in mediating the secretion of antimicrobial substances and preventing excessive inflammatory responses prompted its potential use as a target for the treatment of periodontitis.

Impairment of taste receptors, which are crucial for the host to detect bacteria and mediate regulation oral homeostasis, can lead to overgrowth of pathogenic microorganisms and disease progression. Our study on gingival solitary chemosensory cells (gSCCs) showed that knockout of taste signaling molecules increases bacterial load as well as pathogenicity, ultimately exacerbating periodontitis (Zheng et al., 2019). GSCCs are found in the mouse gingival junctional epithelium, utilizing bitter taste receptors and coupled taste transduction elements to detect bacterial components and modulate immune responses. α-gustducin-null (Gnat3^-/-^) mice which knocked out one of the taste signaling receptors altered the oral microbiome, resulting in greater alveolar bone loss. Meanwhile, topical treatment of bitter denatonium benzoate in wildtype mice could alleviate periodontitis by upregulating the expression of antimicrobial peptides, which was abolished in Gnat3^-/-^ mice. This is coherent with past studies in nasal SCCs (Tizzano et al., 2010; Lee et al., 2014). One plausible hypothesis is that the lack of taste signaling in gSCCs causes insufficient antimicrobial peptides secretion and dysbiosis of microbiota, increasing the risk of periodontitis. These outcomes suggest that taste signaling plays a key role in host oral immune modulation.

## Conclusion

There is now substantial evidence to support that multiple taste receptors are expressed on different cells in the oral cavity to detect bacterial metabolites and distinguish pathogenic from commensal bacteria, playing a critical role in host regulation of oral microbial homeostasis as well as oral health (Zheng et al., 2019). For instance, recent studies in GECs have shown that the expressed bitter taste receptors T2R14 and T2R38 detect various bacterial QSMs or metabolites through different pathways, with the secretion of AMPs to remove pathogenic bacteria (Gil et al., 2015; Medapati et al., 2021b). Functional alterations in taste receptors can also occur as a result of oral microbiota and disease (Zhu et al., 2021). Targeting taste receptors may be a promising alternative therapy to treat oral diseases caused by specific pathogens without antibiotics.

In parallel, numerous studies have described the influence of host genetic polymorphisms on susceptibility to oral diseases (Piekoszewska-Ziętek et al., 2017; Kozak et al., 2020). Similarly, taste receptor SNPs not only determine the distinct taste perception capacities and thus affect food preferences, but also shape the recognition of microbial metabolites, leading to different levels of immune defense in response to specific oral microbes (Chisini et al., 2021). Taste receptor genotypic and phenotypic variations may have potential implications in predicting susceptibility to oral disease and the efficacy of therapy, thereby facilitating the development of personalized treatment based on individual receptor genotypes.

However, current research on the non-gustatory perceptive functions of taste receptors in the oral cavity is still limited, with many crucial questions awaiting further explanations. To name a few, are sour or salt taste receptors in oral likewise engaged in the detection and response to bacterial metabolites? Are there any differences in the transduction pathways between gustatory perception and non-gustatory perception functions? Furthermore, the present understanding of the links and mechanisms between the taste receptor SNPs and oral microbiota as well as diseases is still far from enough. We look forward to further studies on taste receptors to reveal their key role as chemoreceptors in host-pathogen interactions both in oral and systemic settings.

## Author Contributions

Conceptualization: XX, XZ, and MT. Writing original draft: HD, JL, JZ, ZZ. Writing, review, and editing: XP, XDZ, XZ, XX, and MT. funding acquisition: XX and XZ. All authors contributed to the article and approved the submitted version.

## Funding

This work was supported by the National Natural Science Foundation of China (81900995 to XZ, 81771099 to XX), the China Postdoctoral Science Foundation (2020M673266 to XZ), and the Research funding for talents developing, West China Hospital of Stomatology Sichuan University (RCDWJS2020-11 to XZ); the National Institute of Dental and Craniofacial Research (NIDCR) (R01DC028979 to MT), and National Institute on Deafness and Other Communication Disorders (NIDCD) (R01DC016598 to MT).

## Conflict of Interest

The authors declare that the research was conducted in the absence of any commercial or financial relationships that could be construed as a potential conflict of interest.

## Publisher’s Note

All claims expressed in this article are solely those of the authors and do not necessarily represent those of their affiliated organizations, or those of the publisher, the editors and the reviewers. Any product that may be evaluated in this article, or claim that may be made by its manufacturer, is not guaranteed or endorsed by the publisher.
